# Network and pathway‐based analysis of microRNA role in neuropathic pain in rat models

**DOI:** 10.1111/jcmm.14357

**Published:** 2019-05-08

**Authors:** Jia‐Bao Guo, Yi Zhu, Bing‐Lin Chen, Ge Song, Meng‐Si Peng, Hao‐Yu Hu, Yi‐Li Zheng, Chang‐Cheng Chen, Jing‐Zhao Yang, Pei‐Jie Chen, Xue‐Qiang Wang

**Affiliations:** ^1^ Department of Sport Rehabilitation Shanghai University of Sport Shanghai China; ^2^ The Fifth Affiliated Hospital of Zhengzhou University Zhengzhou Henan China; ^3^ School of Medical Technology Xuzhou Medical University Xuzhou Jiangsu China

**Keywords:** biomarker, functional enrichment analysis, miRNA, network analysis, neuropathic pain

## Abstract

The molecular mechanisms underlying neuropathic pain (NP) remain poorly understood. Emerging evidence has suggested the role of microRNAs (miRNAs) in the initiation and development of NP, but the specific effects of miRNAs in NP are largely unknown. Here, we use network‐ and pathway‐based methods to investigate NP‐induced miRNA changes and their biological functions by conducting a systematic search through multiple electronic databases. Thirty‐seven articles meet the inclusion criteria. Venn analysis and target gene forecasting are performed and the results indicate that 167 overlapping target genes are co‐regulated by five down‐regulated miRNAs (rno‐miR‐183, rno‐miR‐96, rno‐miR‐30b, rno‐miR‐150 and rno‐miR‐206). Protein‐protein interaction network analysis shows that 77 genes exhibit interactions, with cyclic adenosine monophosphate (cAMP)‐dependent protein kinase catalytic subunit beta (degree = 11) and cAMP‐response element binding protein 1 (degree = 10) having the highest connectivity degree. Gene ontology analysis shows that these target genes are enriched in neuron part, neuron projection, somatodendritic compartment and nervous system development. Moreover, analysis of Kyoto Encyclopedia of Genes and Genomes reveals that three pathways, namely, axon guidance, circadian entrainment and insulin secretion, are significantly enriched. In addition, rno‐miR‐183, rno‐miR‐96, rno‐miR‐30b, rno‐miR‐150 and rno‐miR‐206 are consistently down‐regulated in the NP models, thus constituting the potential biomarkers of this disease. Characterizing these miRNAs and their target genes paves way for their future use in clinical practice.

## 1 INTRODUCTION

Neuropathic pain (NP) results from damage to or diseases of the somatosensory system and is often accompanied by maladaptive changes in the nervous system.^1^ According to a systematic review of epidemiological studies released in 2014, 7%‐10% of the general population had suffered from NP.^2^ Neuropathic pain has multiple causes, including trauma, metabolic diseases, infection, tumour invasion or neurotoxic chemicals.^3^ The characteristics of chronic NP are complex symptoms, difficult treatments and poor outcomes, which increase the patients' burden and cause anxiety and depression.^4^ To date, the molecular mechanisms on NP have remained poorly understood. Mechanism‐based treatments having an advantage over disease‐ or cause‐based treatments might be the main reason why NP is often treated inadequately or ineffectively.

MicroRNAs (miRNAs) are endogenous small non‐coding RNAs that regulate gene expression by inhibiting protein synthesis.^5^ Evidence suggests that the mechanism for NP involves miRNAs.^6^ Recent studies revealed that miRNAs are highly expressed in the sensory organs of the nervous system, such as dorsal root ganglion (DRG) and spinal dorsal horn (SDH).^7^, ^8^, ^9^ For instance, miR‐183 is significantly down‐regulated in the DRG of a spinal nerve ligation (SNL) rat model. The overexpression of miR‐183 attenuates SNL‐induced mechanical allodynia and this effect is closely connected with the substantial down‐regulation of voltage‐gated sodium channel Nav1.3 and brain‐derived neurotrophic factor (BDNF).^8^ These findings suggest that miRNAs play important roles in the developmental and biological functions of the nervous system for NP.

In 2018, a bioinformatic analysis of miRNAs related to NP used a microarray profile from Gene Expression Omnibus database and included peripheral blood samples from patients with NP after spinal cord injury.^10^ Our study focused on frequently used rat models, such as SNL, spared nerve injury (SNI) and sciatic chronic constriction injury (CCI), to study the miRNA mechanism of NP. We performed a wide literature search on the subject and a subsequent bioinformatics analysis. To the best of our knowledge, this comprehensive bioinformatics analysis is the first to explore the biological functions of miRNAs and identify the potential therapeutic targets for NP.

## 2 MATERIALS AND METHODS

### 2.1 Search strategy

A systematic search was conducted from inception to May 2018. We searched PubMed, EMBASE, Web of Science and Ensco. The following keywords were used: (“MicroRNA*”, “mir*”, “micro RNAs”, “micro RNA”, “micro‐RNAs” or “micro‐RNA”) and (“sciatica*”, “chronic constriction injury”, “CCI”, “partial sciatic nerve injury”, “PNI”, “spinal nerve ligation”, “SNL” “chronic compression dorsal root ganglion”, “CCD”, “spared nerve injury” or “SNI”). The search had no language restrictions. Reference lists of all identified articles were examined. Search strategies for all databases are described in Appendix S1. Search results from the four databases were imported into EndNote (EndNote X7, Bld 7072, Thomson Research Soft, and Stamford) and managed with the entire processes of removing duplicates, reviewing title, abstract and full text.

Studies were identified using the following criteria: (a) type of studies: such as only original articles investigating the role of miRNAs in NP by comparing the animal models of NP to those without pain; (b) type of animal models: rat models of NP including SNL, SNI, CCI, partial sciatic nerve injury and chronic compression of DRG (CCD); (c) type of samples such as nervous tissues (eg, sciatic nerve, DRG, spinal cord and brain) and nervous cells (eg, DRG neurons, microglia and astrocytes); and (d) type of measurements such as miRNA expression assessed by polymerase chain reaction, TaqMan low density array (TLDA) or microarray analysis.

### 2.2 Data extraction

Two researchers (J‐BG and YZ) independently reviewed and extracted data from studies to evaluate their eligibility for inclusion. We extracted data, including the first author, publication year, country, experimental design (eg, experimental models, region used) and information on miRNAs (eg, expression change, target genes and functions). Any discrepancy for selection and extraction was resolved by a third researcher (B‐LC).

### 2.3 Bioinformatics analysis

To examine the functional roles of miRNAs, we predicted the target genes of miRNAs by using the TargetScan software (http://www.targetscan.org/). Venn diagram analysis showing the number of overlapping miRNAs and targets was based on Functional Enrichment analysis tool (FunRich; http://www.funrich.org/). The minimum fold value for up‐regulated and down‐regulated miRNAs in the matrix table is 2. A protein‐protein interaction (PPI) network was used to further understand the correlations between the overlapping targets of differentially expressed miRNAs. Protein‐protein interaction data sources were obtained from the String database (http://string-db.org/),^11^ and maps were drawn with Cytoscape software v.3.6.0.^12^ A PPI confidence score of >0.4 was used for the construction of the PPI network and cytoscape software was utilized to obtain the results of gene ontology (GO) annotation and Kyoto Encyclopedia of Genes and Genomes (KEGG) analyses. Moreover, GO annotation was analysed to explore the functional roles of putative targets regarding biological process, cellular component and molecular functions. A Fisher exact test *P*‐value <0.05 was used to identify significantly targeted pathways. The enrichment results were presented using GraphPad Prism 7.0.1 (GraphPad Software Inc, La Jolla, CA).

## 3 RESULTS

**Figure 1:**
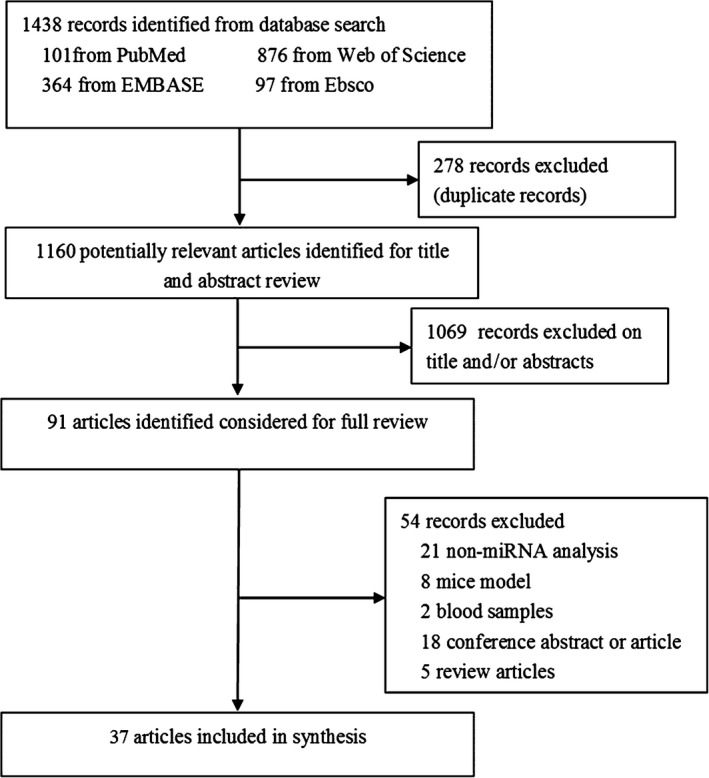
Flow chart of the study selection procedure (for details of study identification)

We identified 1438 articles through electronic search. After the exclusion of duplicate records, 1160 articles remained. The researchers then examined the title and abstract and 91 articles were identified for further full‐text review. Finally, 37 articles fulfilled the eligibility criteria and were included.^7^, ^8^, ^9^, ^11^, ^12^, ^13^, ^14^, ^15^, ^16^, ^17^, ^18^, ^19^, ^20^, ^21^, ^22^, ^23^, ^24^, ^25^, ^26^, ^27^, ^28^, ^29^, ^30^, ^31^, ^32^, ^33^, ^34^, ^35^, ^36^, ^37^, ^38^, ^39^, ^40^, ^41^, ^42^, ^43^, ^44^ Among these studies, six^11^, ^12^, ^13^, ^14^, ^15^, ^16^ were about miRNA profiles and 32^7^, ^8^, ^9^, ^16^, ^17^, ^18^, ^19^, ^20^, ^21^, ^22^, ^23^, ^24^, ^25^, ^26^, ^27^, ^28^, ^29^, ^30^, ^31^, ^32^, ^33^, ^34^, ^35^, ^36^, ^37^, ^38^, ^39^, ^40^, ^41^, ^42^, ^43^, ^44^ were on miRNA experimental verification, of which one^16^ was conducted miRNA profile and experimental verification. The flowchart of the study selection procedure is detailed in Figure 1.

### 3.1 Study characteristics

The main characteristics of the included articles are shown in Tables 1 and 2. As shown in Table 1, six articles^11^, ^12^, ^13^, ^14^, ^15^, ^16^ examined the miRNA changes in NP rat models through TLDA card or microarray analysis. The numbers of the significantly dysregulated miRNAs in these seven studies varied from 1 to 22. In the earliest study,^11^ the authors investigated and compared the miRNA expression profile in the spinal cord of rats with CCI with that of sham‐operated rats. The results indicated six down‐regulated miRNAs. In the latest study,^16^ miRNA changes in anterior cingulate cortex (ACC) after CCI were examined by using microarray. Nine miRNAs were significantly up‐regulated and 11 were significantly down‐regulated.

**Table 1:** Expression profiles of miRNAs

Article, Year	Country	Expression	miRNAs	Experimental models	Method
Brandenburger et al, 2012^11^	USA	Down	mmu‐miR‐30b, mmu‐miR‐100, mmu‐miR‐10a, mmu‐miR‐99a, mmu‐miR‐582‐3p, mmu‐miR‐720	Spinal cord from CCI rats	Microarray
Arai et al, 2013^12^	Japan	Up	hsa‐miR‐22, has‐miR‐338	Hippocampus from CCI rats	TLDA
		Down	mmu‐miR‐124, mmu‐miR‐132, mmu‐miR‐151‐3p, mmu‐miR‐186, mmu‐miR‐187, mmu‐miR‐204, mmu‐miR‐210, mmu‐miR‐25, mmu‐miR‐27a, mmu‐miR‐30e, mmu‐miR‐34c, mmu‐miR‐448, mmu‐miR‐449a, mmu‐miR‐488, mmu‐miR‐668, mmu‐miR‐92a, mmu‐miR‐98, rno‐miR‐1		
Genda et al, 2013^13^	China	Up	mmu‐miR‐539, rno‐miR‐381, mmu‐miR‐323‐3p	SDH from CCI rats	TLDA
		Down	mmu‐miR‐22, mmu‐miR‐496, mmu‐miR‐151‐3p, mmu‐miR‐24‐2, mmu‐miR‐324‐5p, rno‐miR‐345‐3p, mmu‐miR‐127, mmu‐miR‐125b‐5p, mmu‐miR‐221, mmu‐miR‐296‐5p, rno‐miR‐377, mmu‐miR‐365, mmu‐miR‐598, mmu‐miR‐7a, mmu‐miR‐101b, mmu‐miR‐29b, rno‐miR‐336, has‐miR‐493‐3p, mmu‐miR‐322, mmu‐miR‐21, mmu‐miR‐27b, rno‐miR‐632		
Li et al, 2013^14^	China	Up	rno‐miR‐341	DRG from CCI rats	Microarray
		Down	rno‐miR‐203, rno‐miR‐181a‐1, rno‐miR‐541	SDH from CCI rats	
Chang et al, 2017^15^	China	Up	rno‐miR‐146b, rno‐miR‐21, rno‐miR‐21‐3p, rno‐miR‐221, rno‐miR‐222, rno‐miR‐31, rno‐miR‐339‐3p, rno‐miR‐344b‐1‐3p, rno‐miR‐3566, rno‐miR‐3574, rno‐miR‐3596d, rno‐miR‐466b‐1, rno‐miR‐466b‐2, rno‐miR‐466c	DRG from SNL rats	Microarray
		Down	rno‐miR‐122, rno‐miR‐125b‐3p, rno‐miR‐214, rno‐miR‐297, rno‐miR‐32‐3p, rno‐miR‐351‐3p, rno‐miR‐3560, rno‐miR‐3584‐5p, rno‐miR‐3588, rno‐miR‐363‐5p, rno‐miR‐466b, rno‐miR‐466c, rno‐miR‐466d, rno‐miR‐664‐1‐5p, rno‐miR‐664‐2‐5p, rno‐miR‐665, rno‐miR‐668, rno‐miR‐672, rno‐miR‐92a‐2‐5p, rno‐miR‐99b‐3p		
Ding et al, 2017^16^	China	Up	rno‐miR‐493, rno‐miR‐205, rno‐miR‐203, rno‐miR‐194, rno‐miR‐380, rno‐miR‐21, rno‐miR‐341, rno‐miR‐221, rno‐miR‐499	ACC from CCI rats	Microarray/qRT‐PCR
		Down	rno‐miR‐192, rno‐miR‐144, rno‐miR‐500, rno‐miR‐340‐5p, rno‐miR‐327, rno‐miR‐296, rno‐miR‐539, rno‐miR‐505, rno‐miR‐214, rno‐miR‐129, rno‐miR‐223		

Abbreviations: ACC, anterior cingulate cortex; CCI, sciatic chronic constriction injury; DRG, dorsal root ganglion; qRT‐PCR, quantitative real‐time polymerase chain reaction; SDH, spinal dorsal horn; SNL, spinal nerve ligation; TLDA, TaqMan low density array.

**Table 2:** Experimentally verified miRNAs

Article, Year	Country	Models	Region	miRNAs	Expression change	Target gene(s)	Functions
Aldrich et al, 2009^17^	USA	SNL	DRG	rno‐miR‐96/183	Down	NR	NR
Favereaux et al, 2011^18^	France	SNL	Spinal cord, spinal neuron	rno‐miR‐103	Down	Cav1.2	Neuronal excitability
Sakai et al, 2013^19^	Japan	SNL/CCI	DRG, DRG neuron	rno‐miR‐21	Up	IL‐1β	Neuroinflammation
Sakai et al, 2013^20^	Japan	SNL	DRG neuron	rno‐miR‐7a	Down	Scn2b	Neuronal excitability
Shi et al, 2013^21^	China	SNL	SDH, microglia	rno‐miR‐195	Up	ATG14	Neuroinflammation
Lin et al, 2014^8^	Taiwan	SNL	DRG	rno‐miR‐183	Down	Nav1.3, BDNF	Neuronal excitability
Yang et al, 2016^22^	China	SNL/CCI	DRG, PC12 cell	rno‐miR‐206	Down	RASA1	Neuronal plasticity
Su et al, 2017^23^	China	SNL	Spinal cord, DRG neuron	rno‐miR‐30b	Down	Nav1.3	Neuronal excitability
Xu et al, 2017^24^	China	SNL	DRG, DRG neuron	rno‐miR‐143	Down	DNM3a	DNA methylation
Yan et al, 2018^25^	China	SNL	SDH, microglia	rno‐miR‐32‐5p	Up	Dusp5	Neuroinflammation
Leinders et al, 2016^7^	USA	SNI	SDH, DRG, Microglia	rno‐miR‐132‐3p	Up	GluA1, GluA2	Neuronal plasticity
Shao et al, 2016^26^	China	SNI	DRG, PC12 cell	rno‐miR‐30b	Down	Nav1.7	Neuronal excitability
Chen et al, 2014^27^	China	CCI	DRG	rno‐miR‐96	Down	Nav1.3	Neuronal excitability
Li et al, 2015^28^	China	CCI	SDH, PC12 cell	rno‐miR‐203	Down	Rap1a	Neuronal plasticity
Neumann et al, 2015^29^	Germany	CCI	Sciatic nerve	rno‐miR‐1	Down	BDNF, Cx43	Neuroinflammation
Tan et al, 2015^30^	China	CCI	Spinal cord, microglia	rno‐miR‐155	Up	SOCS1	Neuroinflammation
Wang et al, 2015^31^	China	CCI	Spinal cord	rno‐miR‐19a	Up	SOCS1	Neuroinflammation
Zhang et al, 2015^32^	China	CCI	DRG, DRG neuron	rno‐miR‐141	Down	HMGB1	Neuroinflammation
Li et al, 2016^33^	China	CCI	Spinal cord, microglia	rno‐miR‐218	Up	SOCS3	Neuroinflammation
Pang et al, 2016^34^	China	CCI	Spinal cord	rno‐miR‐145	Down	RREB1, p‐AKT	Neuroinflammation
Xia et al, 2016^35^	China	CCI	Spinal cord, microglia	rno‐miR‐221	Up	SOCS1	Neuroinflammation
Sun et al, 2017^9^	China	CCI	DRG, PC12 cell	rno‐miR‐206	Down	BDNF	Neuroinflammation
Ding et al, 2017^16^	China	CCI	ACC	rno‐miR‐539	Down	NR2B	Neuronal plasticity
Xie et al, 2017^36^	China	CCI	SDH, PC12 cell	rno‐miR‐183	Down	mTOR	Neuroinflammation
Yan et al, 2017^37^	China	CCI	SDH, microglia	rno‐miR‐200b/429	Down	ZEB1	Neuroinflammation
Yan et al, 2017^38^	China	CCI	SDH, microglia	rno‐miR‐93	Down	STAT3	Neuroinflammation
Zhao et al, 2017^39^	China	CCI	SDH	rno‐miR‐137	Down	TNFAIP1	Neuroinflammation
Ji et al, 2018^40^	China	CCI	SDH, microglia	rno‐miR‐150	Down	TLR5	Neuroinflammation
Jin et al, 2018^41^	China	CCI	SDH, microglia	rno‐miR‐544	Down	STAT3	Neuroinflammation
Shi et al, 2018^42^	China	CCI	DRG	rno‐miR‐183‐5p	Down	TREK‐1	Neuronal excitability
Xia et al, 2018^43^	China	CCI	SDH	rno‐miR‐381	Down	HMGB1	Neuroinflammation
Yan et al, 2018^44^	China	CCI	SDH, microglia	rno‐miR‐150	Down	ZEB1	Neuroinflammation

Abbreviations: ACC, anterior cingulate cortex; AMPA receptor subunit, GluA1 and GluA2; ATG14, autophagy related gene 14; BDNF, brain‐derived neurotrophic factor; CCI, sciatic chronic constriction injury; Cx43, Connexin 43; DNM3a, DNA methyltransferase 3a; DRG, dorsal root ganglion; Dusp5, dual‐specificity phosphatase 5; HMGB1, high mobility group box 1; mTOR, mammalian target of rapamycin; NR2B, N‐methyl‐D‐aspartate receptors 2B; NR, not reported; p‐AKT, phosphorylated‐protein kinase B; Rap1a, Ras‐related protein Rap‐1A; RASA1, RAS p21 protein activator 1; RREB1, Ras responsive element binding protein 1; SDH, spinal dorsal horn; SNI, spared nerve injury; SNL, spinal nerve ligation; SOCS, suppressor of cytokine signalling; STAT3, signal transducer and activator of transcription 3; TLR5, toll‐like receptor 5; TNFAIP1, tumour necrosis factor alpha‐induced protein 1; TREK‐1, TWIK‐related K^+^ channel 1; ZEB1, zinc finger E box binding protein 1.

Thirty‐two articles^7^, ^8^, ^9^, ^16^, ^17^, ^18^, ^19^, ^20^, ^21^, ^22^, ^23^, ^24^, ^25^, ^26^, ^27^, ^28^, ^29^, ^30^, ^31^, ^32^, ^33^, ^34^, ^35^, ^36^, ^37^, ^38^, ^39^, ^40^, ^41^, ^42^, ^43^, ^44^ experimentally verified that 27 miRNAs may promote the regulation of NP (Table 2). Table 1 shows three NP models, namely, SNL, SNI and CCI and their comparison with sham operation. Regarding the tissues analysed, we verified that miRNA expression was changed in nervous tissues such as sciatic nerve, DRG, SDH and ACC and nervous cells such as DRG neurons and microglia. For instance, 3.13% (1/32) of the studies evaluated the miRNA expression in sciatic nerve, 34.38% (11/32) in DRG, 59.38% (19/32) in spinal cord and 3.13% (1/32) in brain regions. Furthermore, several miRNAs were changed in DRG and spinal cord.^7^, ^29^, ^39^ According to the studies included, 20 miRNAs^8^, ^9^, ^16^, ^17^, ^18^, ^20^, ^22^, ^23^, ^24^, ^26^, ^27^, ^28^, ^29^, ^32^, ^34^, ^36^, ^37^, ^38^, ^39^, ^40^, ^41^, ^42^, ^43^, ^44^ were commonly down‐regulated, of which rno‐miR‐183, rno‐miR‐96, rno‐miR‐30b, rno‐miR‐150 and rno‐miR‐206 were reported twice or more. Moreover, eight miRNAs^7^, ^19^, ^21^, ^25^, ^30^, ^31^, ^33^, ^35^ were up‐regulated. However, none of the studies focused on the same miRNA. We then categorized the functions of these NP‐related miRNAs as neuroinflammation, neuronal excitability, neuronal plasticity and DNA methylation.

### 3.2 Target prediction and Venn diagram analysis

**Figure 2:**
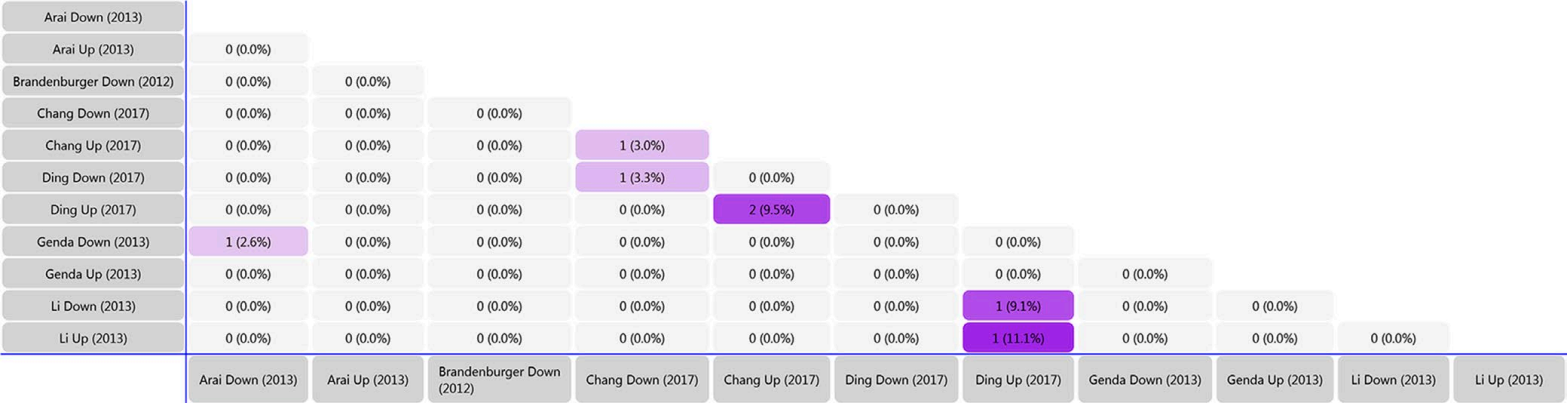
Matrix table analysis. The table is based on the miRNAs identified in Table 1 and shows the number and percentage of co‐regulated miRNAs. The minimum fold value for up‐regulated and down‐regulated miRNAs is 2. miRNAs, microRNAs

**Figure 3:**
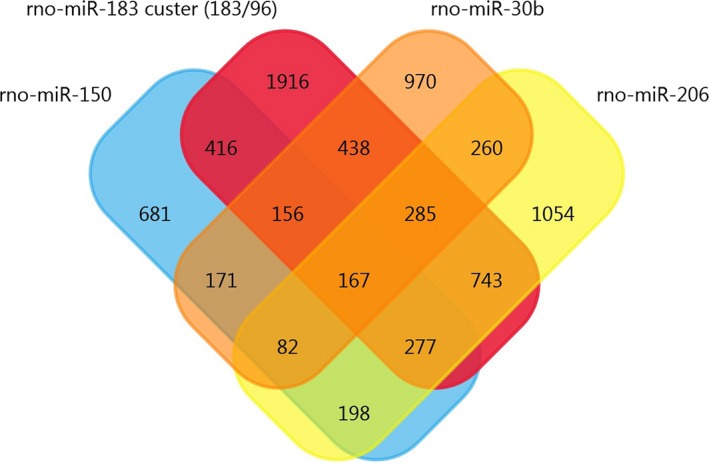
Venn diagram analysis. Overlapping target genes of rno‐miR‐183/96, rno‐miR‐30b, rno‐miR‐150 and rno‐miR‐206. These five down‐regulated miRNAs were identified in Table 2 and have been observed in two or more studies. miR‐183 and miR‐96 belong to the miR‐183 cluster, so we combined their target genes to easily create the Venn diagram. miRNAs, microRNAs

Using the array data from Table 1, we generated a matrix table with a FunRich tool (Figure 2) and it shows the number and percentage of co‐regulated miRNAs through pair‐wise comparison. In the matrix table, we found that rno‐miR‐221, rno‐miR‐21 and rno‐341 were up‐regulated in two or more studies, whereas mmu‐miR‐151‐3p and rno‐miR‐214 were down‐regulated in two or more studies. Target Scan software was subsequently used to forecast the target genes of miRNAs and Venn diagrams were drawn. We found 20 overlapping target genes in the three up‐regulated miRNAs (rno‐miR‐221, rno‐miR‐21 and rno‐341), but none in the two down‐regulated miRNAs (mmu‐miR‐151‐3p and rno‐miR‐214) (Figures S1 and S2). By analysing Table 2, we found that 167 overlapping target genes are present in the five down‐regulated miRNAs (rno‐miR‐183, rno‐miR‐96, rno‐miR‐30b, rno‐miR‐150 and rno‐miR‐206) (Figure 3). The miR‐183 cluster comprises miR‐183, miR‐96 and miR‐182 and shares the same sequence homology. Therefore, we combined the target genes of rno‐miR‐183 and rno‐miR‐96 for Venn diagrams analysis.

### 3.3 PPI network analysis

**Figure 4:**
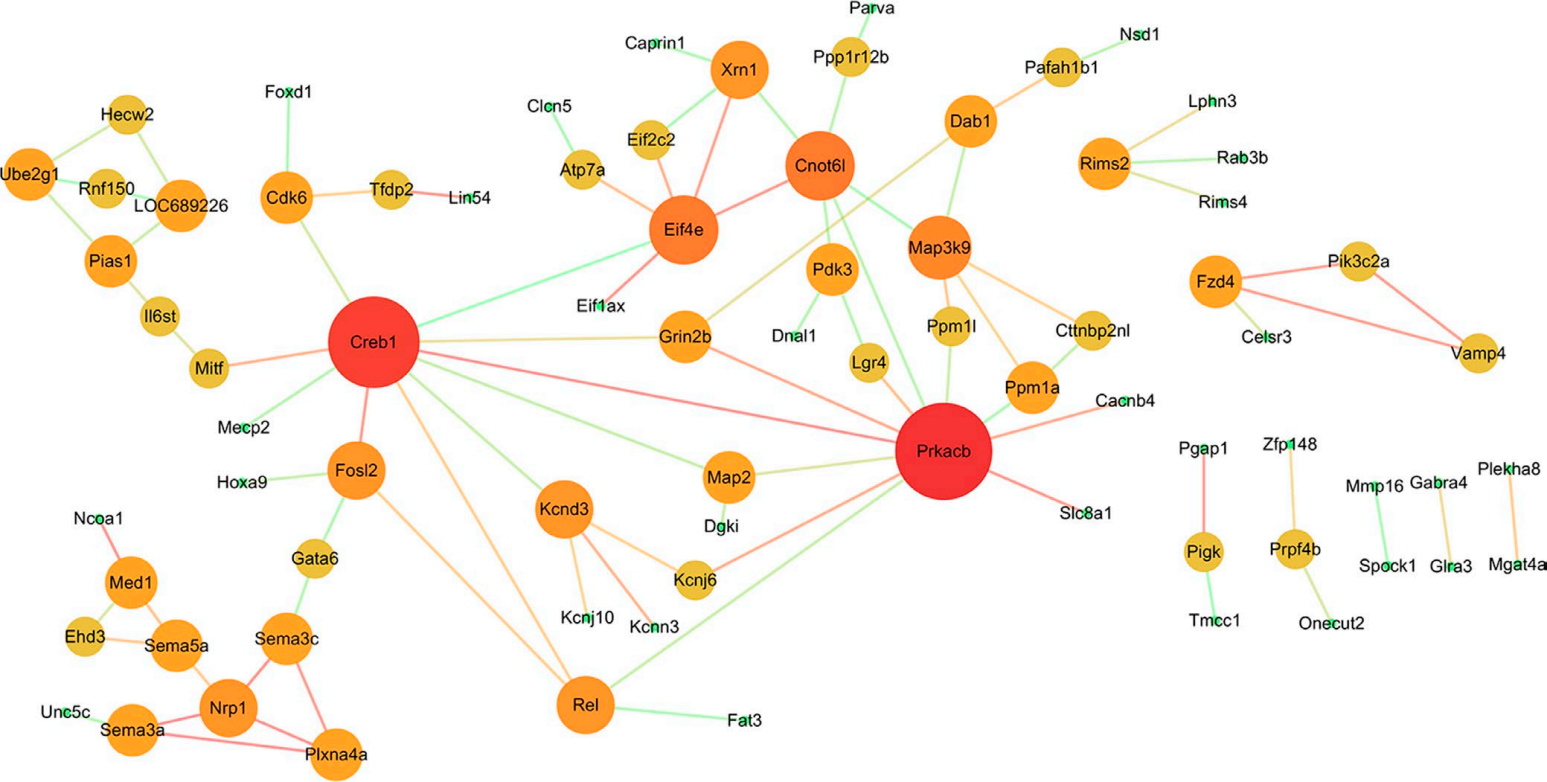
Protein‐protein interaction network analysis. A total of 167 overlapping target genes were obtained from Figure 3. The sizes and colours of each node represent the degree of functional connection with these genes. The colours of each edge indicate the strength of data support as evaluated by combined scores. A low value is represented by small sizes and bright colours in the map and a high value is represented by large sizes and dark colours

**Figure 5:**
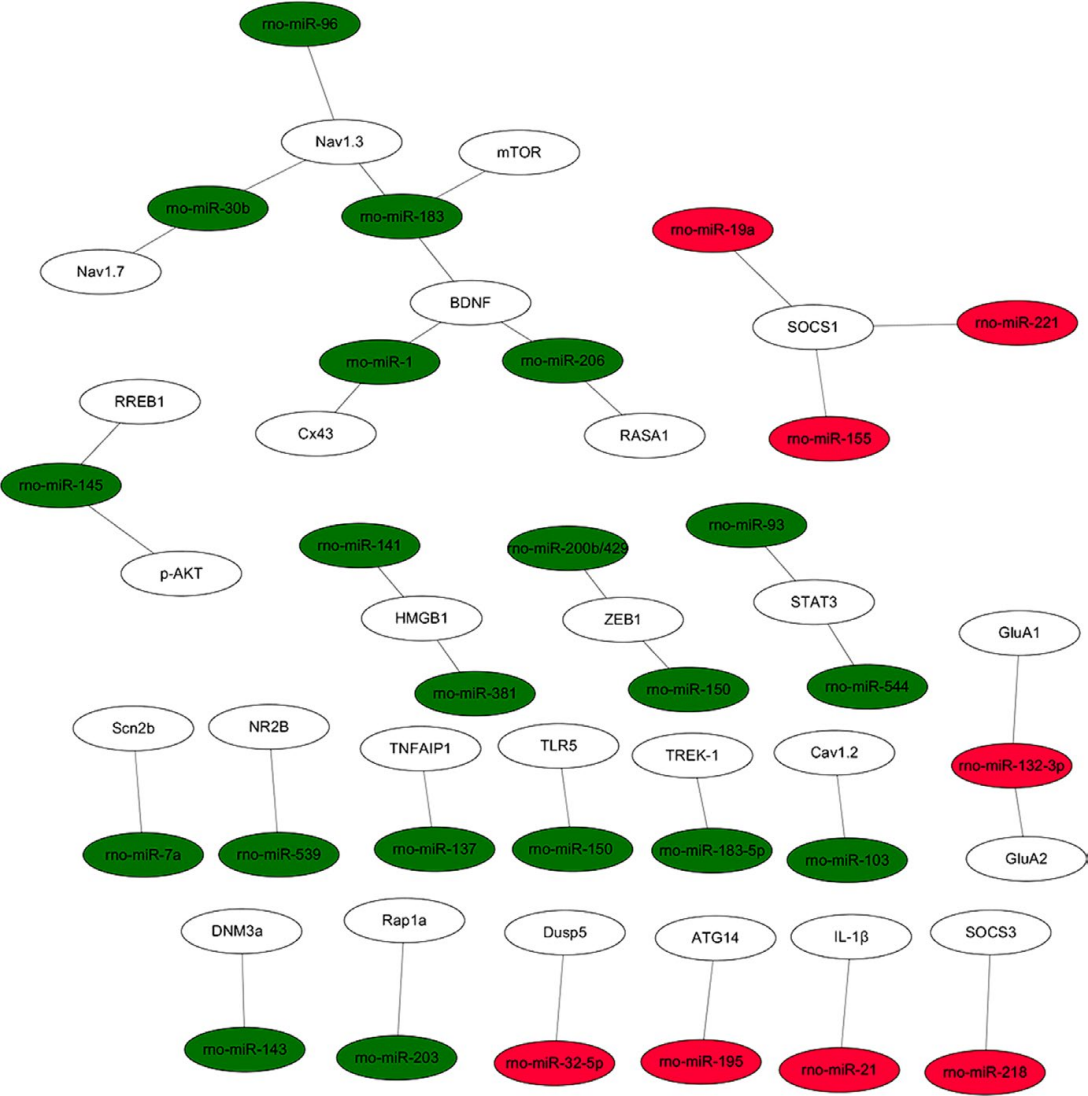
Dysregulated miRNA‐target gene network. The network is based on the dysregulated miRNAs and their target genes identified in Table 2. Green colours represent down‐regulated miRNAs and red colours represent up‐regulated miRNAs. miRNAs, microRNAs

The PPI data of 167 overlapping target genes were obtained from String database and the network was displayed by using Cytoscape software. The results of PPI analysis are shown in Figure 4. A total of 77 genes exhibited interactions. The sizes and colours of each node represent the degree of functional connection with the 167 genes. The colours of each edge indicate the strength of data support, as evaluated by combined scores. A low value is represented by small sizes and bright colours in the map and a high value is represented by large sizes and dark colours. The cyclic adenosine monophosphate (cAMP)‐response element binding protein1 (CREB1) and cAMP‐dependent protein kinase catalytic subunit beta (PRKACB) were the two largest and darkest nodes in the network. Hence, PRKACB (degree = 11) and CREB1 (degree = 10) exhibited the highest connectivity degree. Furthermore, we mapped the miRNAs of Table 2 to their target genes (Figure 5). Three down‐regulated miRNAs (rno‐miR‐183, rno‐miR‐96 and rno‐miR‐30b) directly targeted Nav1.3 and BDNF. According to the analysis of the up‐regulated miRNA and their target gene network, the suppressor of cytokine signalling‐1 (SOCS1) is a direct target of rno‐miR‐155, rno‐miR‐19a and rno‐miR‐221.

### 3.4 Functional enrichment analysis

**Figure 6:**
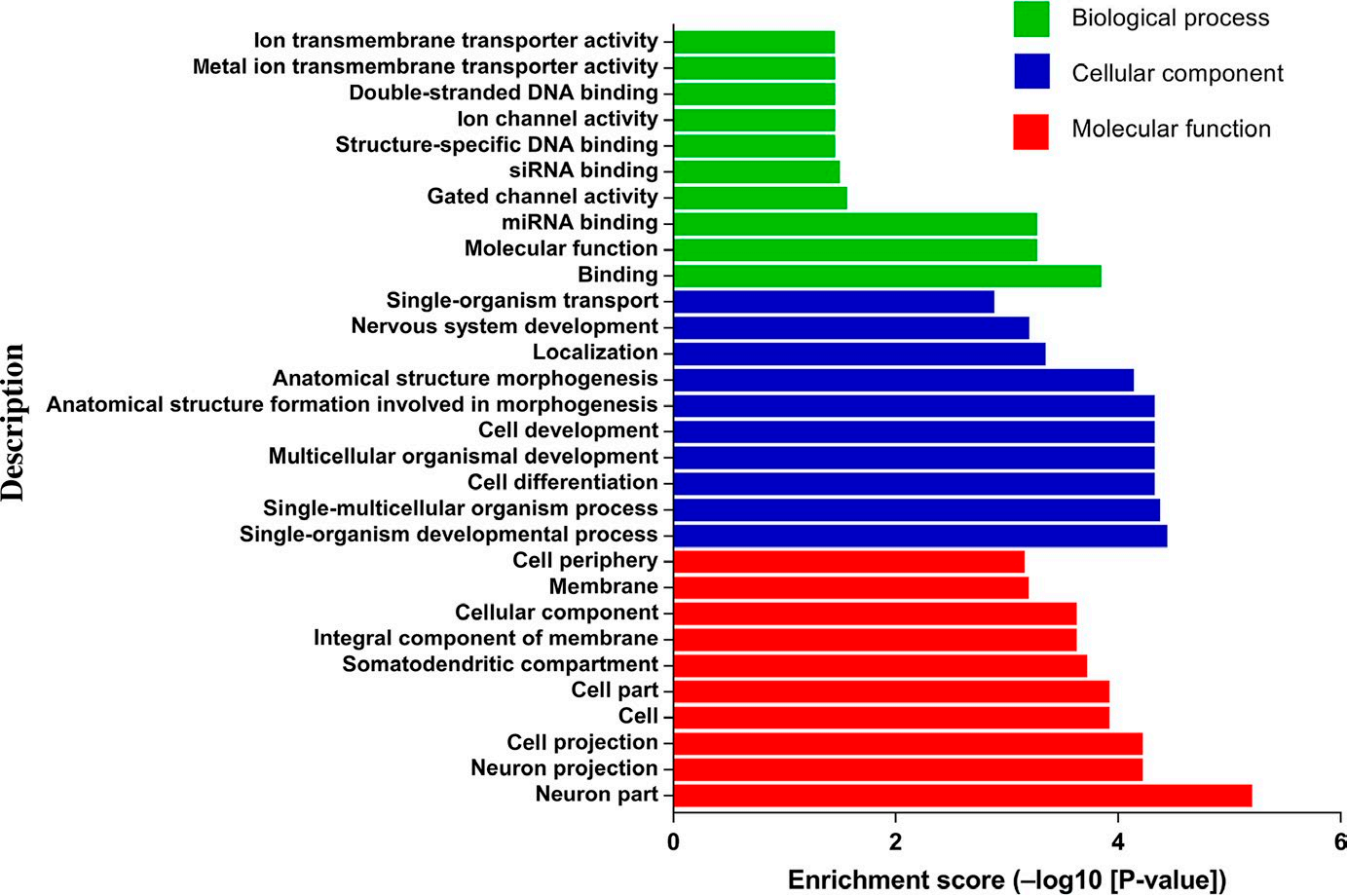
GO annotation enrichment analysis. The vertical axis is the description of GO terms and the horizontal axis is the enrichment score (–log10[*P*‐value]) of the pathways; log10[*P*‐value] is the logarithm of the *P*‐value and *P* < 0.05 was considered significant. GO, gene ontology

We analysed 167 overlapping target genes from five down‐regulated miRNAs (rno‐miR‐183, rno‐miR‐96, rno‐miR‐30b, rno‐miR‐150 and rno‐miR‐206), as shown in Figure 3. A total of 128 GO terms were significantly enriched (*P < *0.05) and the top 10 high enrichment score pathways are shown in Figure 6. The results of the GO analysis comprised biological processes (eg, single‐organism developmental process, GO: 0044767), cellular components (eg, neuron part, GO: 0097458) and molecular functions (eg, binding, GO: 0005488). Many of the enriched terms were associated with the nervous system; such terms included neuron part (GO: 0097458), neuron projection (GO: 0043005), somatodendritic compartment (GO: 0036477) and nervous system development (GO: 0007399). Moreover, KEGG analysis showed that three pathways, namely, axon guidance, circadian entrainment and insulin secretion pathways, were significantly enriched (*P < *0.05, Table S1).

## 4 DISCUSSION

**Figure 7:**
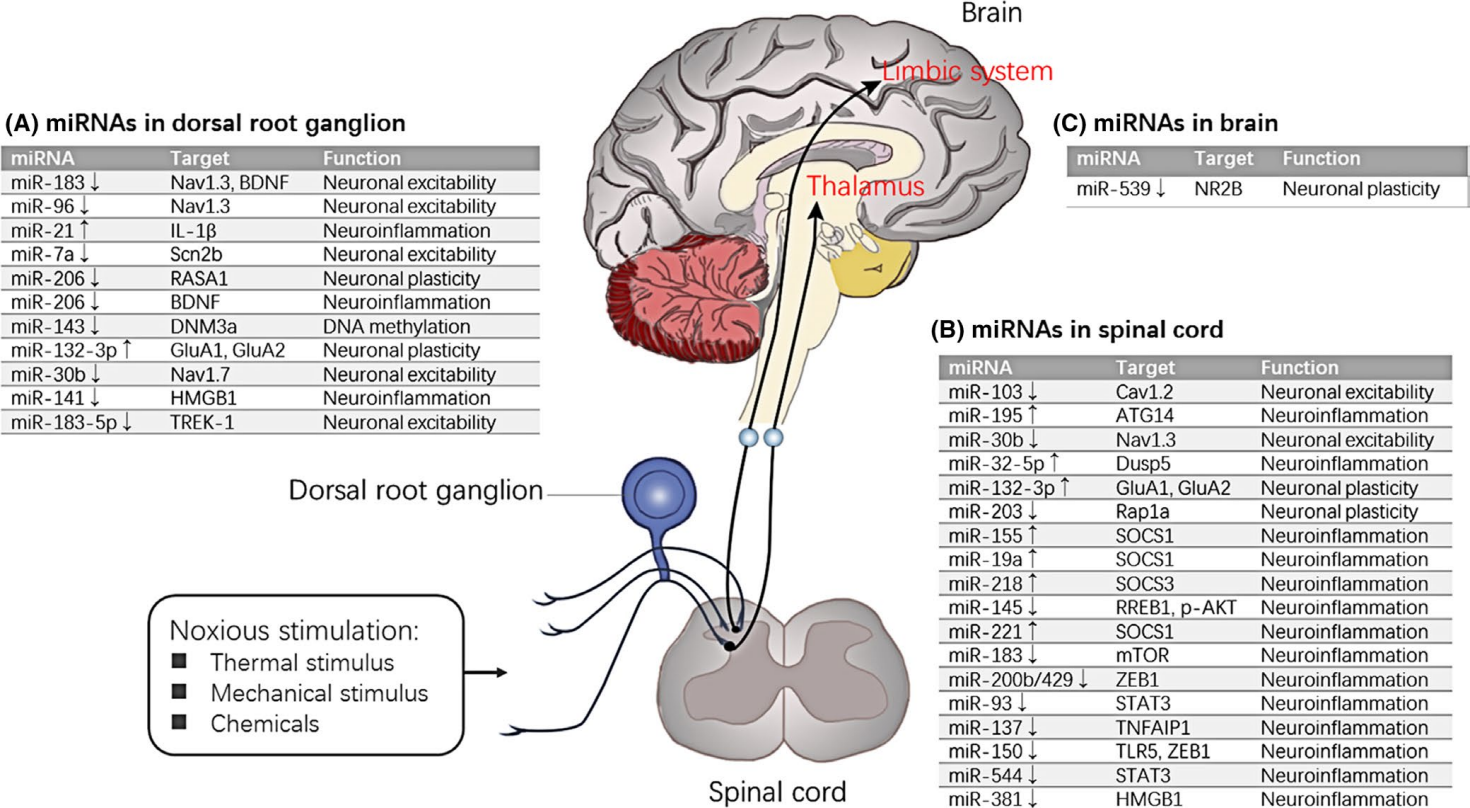
Ascending NP pathway and miRNA regulation of pain genes. Noxious stimulation reaches SDH though afferent nerve fibre. SDH is the site responsible for integrating and processing information of sensory inputs and carries the output to the brain by several pathways. The thalamus and limbic system are important sites in the brain for ascending the NP pathway. miRNA modulation is reflected in DRG, SDH and several brain areas. A, miRNAs, their target genes and functions in DRG. B, miRNAs, their target genes and functions in spinal cord. C, miRNAs, their target genes and functions in brain. miRNAs, microRNAs; NP, neuropathic pain; DRG, dorsal root ganglion; SDH, spinal dorsal horn BDNF, brain‐derived neurotrophic factor; RASA1, RAS p21 protein activator 1; DNM3a, DNA methyltransferase 3a; AMPA receptor subunit, GluA1 and GluA2; HMGB1, High mobility group box 1; TREK‐1, TWIK‐Related K+ Channel 1; NR2B, N‐methyl‐D‐Aspartate receptors 2B; ATG14, Autophagy Related Gene 14; Dusp5, Dual‐specificity phosphatase 5; Rap1a, Ras‐related protein Rap‐1A; SOCS, Suppressor of cytokine signaling; RREB1, Ras responsive element binding protein 1; p‐AKT, phosphorylated‐protein kinase B; mTOR, mammalian target of rapamycin; ZEB1, Zinc finger E box binding protein 1; STAT3, signal transducer and activator of transcription 3; TNFAIP1, tumor necrosis factor alpha‐induced protein 1; TLR5, toll‐like receptor 5

In this study, we focused on miRNA expression in DRG, SDH and ACC. These regions play important roles in the somatosensory pathway from primary sensory neurons to the central nervous system in NP. DRG neurons receive nociceptive afferents carrying peripheral inputs (eg, heat, cold, pressure and chemicals) and transmit information to second‐order neurons mostly in SDH. After the integration and processing of the sensory inputs in SDH, the outputs from the spinal networks are carried to the higher cortical centres.^45^ Anterior cingulate cortex is a crucial brain region of the limbic system and is associated with the anticipation of pain and attention to pain.^46^, ^47^ MicroRNAs in NP are involved in neuroinflammation, neuronal excitability, neuronal plasticity and DNA methylation (Figure 7). Several studies focused on neuronal excitability mechanisms. Peripheral nerve injury induces the hyperexcitability of injured afferent neurons, thereby contributing to ectopic discharge.^48^ Voltage‐gated ion channels are the molecular bases of generating action potential^49^; such channels contain voltage‐gated calcium channels, such as Cav1.2 and voltage‐gated sodium channels, such as Nav1.3 and Nav1.7. Favereaux et al^18^ demonstrated that miR‐103 bidirectionally regulates the expression of Cav1.2‐comprising L‐type calcium channel (Cav1.2‐LTC) formed by three subunits, namely, Cav1.2‐α1, Cav1.2‐α2δ1 and Cav1.2‐β1. The expression of miR‐103 in the SDH significantly decreased and the expression levels of the three Cav1.2 subunits increased in SNL rats compared with those in sham rats. However, the high expression of Cav1.2 subunit mRNAs was reversed after receiving intrathecal injection of miR‐103. Nav1.3 is a direct target gene of the three miRNAs (rno‐miR‐183, rno‐miR‐96 and rno‐miR‐30b). Shao et al^26^ verified that miR‐30b regulated the expression of Nav1.7 in DRG through miR‐30b overexpression or knock‐down following SNI. Su et al^23^ focused on miR‐30b and Nav1.3 in SNL‐induced NP; the result showed that miR‐30b can suppress the expression of Nav1.3 in DRG neurons and spinal cord in SNL rats. Overall, these miRNAs may play a role in neuronal excitability mechanisms of NP by silencing important target voltage‐gated ion channels.

Studies on the accurate functional roles of differential miRNAs are insufficient; in this regard, we examined the target genes of the included miRNAs in the present work. The analyses of the PPI network and functional enrichment were conducted to determine the functional roles of the overlapping target genes in rat models of NP. The PPI network analysis identified the highest connectivity degree between PRKACB and CREB1. Cyclic adenosine monophosphate‐dependent protein kinase catalytic subunit beta is an important component of the activity of cAMP‐dependent protein kinase A. One of the best‐characterized roles of PRKACB in the hippocampus is regulation of synaptic excitability and long‐term potentiation.^50^ A recent work demonstrated the occurrence of inflammation‐induced long‐term potentiation of nociceptive transmission in ACC.^51^ Cyclic adenosine monophosphate‐response element binding protein functions closely with brain‐related miRNAs to mediate neuronal gene expression.^52^ Cyclic adenosine monophosphate‐response element binding protein is a nuclear transcription factor that is activated at the serine 133 site through phosphorylation.^53^ Cyclic adenosine monophosphate‐response element binding protein and signalling cascade participate in central sensitization and pathogenesis of NP.^54^, ^55^ The phosphorylation level of CREB increases in the spinal cord of rats in multiple NP models.^56^, ^57^ cAMP‐CREB and mitogen activated protein kinase (MAPK)‐CREB signalling pathways regulate the phosphorylation of CREB.^58^, ^59^

Gene ontology annotation revealed many enriched terms that focused on the functions of the nervous system; such terms included neuron part, neuron projection, somatodendritic compartment and nervous system development. The KEGG analysis indicated the relation of axon guidance, circadian entrainment and insulin secretion to NP. Axon guidance plays crucial role in establishing neuronal circuitry and can regulate the translation of local messenger RNA (mRNA) by axon guidance cues.^60^ Previous studies speculated that preventing the development of NP can be partly achieved using axon guidance cues, such as semaphorin‐3A, netrin‐1 and ephrin‐B.^61^, ^62^, ^63^ For example, intrathecal administration of semaphorin‐3A in the spinal cord of CCI rat model would attenuate NP‐related behaviour on mechanical allodynia and heat hyperalgesia. The EphrinB‐EphB receptor suppresses NP by controlling neural excitability and synaptic plasticity in DRG and SDH.^62^ With regard to circadian entrainment, NP exhibited apparent pain rhythm, which is characterized with hypnalgia. The miR‐183/96/182 cluster plays important role in sensory neural biology and the expression levels of miR‐182 and miR‐96 showed circadian rhythm.^64^ Emerging evidence suggests that miRNAs may contribute to the post‐transcriptional regulation of clock genes in NP.^65^, ^66^ In addition, Xia^67^ attested the direct relation of N‐methyl‐D‐Aspartate receptors containing N‐methyl‐D‐Aspartate receptor 2B (NR2B)‐CREB regulated transcription coactivator 1 (CRTC1)‐CREB signalling pathway to the circadian rhythm mediated by the suprachiasmatic nucleus. Previous studies indicated that insulin secretion suppresses spinal nociceptive processing after nerve injury.^68^ In this study, we provide a comprehensive and systematic framework for investigating the potential biological functions associated with NP. This work highlights a broad search strategy without any publication date or language restrictions and offers significant sources for further analysis. In addition, a high coverage of miRNAs associated with NP was collected to explore the interaction of their target genes by using network and pathway‐based analyses. The results might provide a comprehensive view of the molecular mechanisms underlying NP. Although the results of the bioinformatic analysis may be affected by heterogeneity from differences in the study design, such as different NP models and nerve tissues, combining network analysis with pathway analysis is able to be more robust for possible false positives resulted from various miRNAs in different studies.

In summary, our study provides an important step for describing the NP sensitivity of miRNA expression and the target gene regulatory consequences of related expression changes. Bioinformatics analysis elucidates the functional roles of five miRNAs (rno‐miR‐183, rno‐miR‐96, rno‐miR‐30b, rno‐miR‐150 and rno‐miR‐206) and their targets involved in the known relevant pathways for NP. They may serve as potential biomarkers and novel strategies for prevention and treatment of NP following peripheral nerve injury. Further studies are necessary to confirm the involvement of the five miRNAs in different stages of NP and translate the findings to clinical practice.

## CONFLICTS OF INTEREST

The authors declare that they have no competing interests.
