# Supplementary material for: Network and pathway‐based analysis of microRNA role in neuropathic pain in rat models
Source: J Cell Mol Med. 2019 May 8;23(7):4534–44. doi: 10.1111/jcmm.14357 (PMC6584487; doi:10.1111/jcmm.14357)
Supplement: Supplementary file 1 [file JCMM-23-4534-s001.docx]

**Appendix S1: Search strategies for all databases**

**1. Search Strategy for Pubmed**

#1 MicroRNAs[Mesh Terms] OR "microRNA*"[Text Word] OR "mir*"[Text Word] OR "micro RNA"[Text Word] OR "micro RNAs"[Text Word] OR "micro-RNA"[Text Word] OR "micro-RNAs"[Text Word]

#2  Sciatica[Mesh Terms] OR "sciatica*"[Text Word] OR "ischial*"[Text Word] OR "sciatic neuropathy"[Text Word] OR "sciatic pain"[Text Word] OR "sciatic nerve palsy"[Text Word] OR "sciatic nerve diseases"[Text Word] OR "sciatic neuritis"[Text Word] OR "chronic constriction injury"[Text Word] OR "CCI"[Text Word] OR "partial sciatic nerve injury"[Text Word] OR "PNI"[Text Word] OR "spinal nerve ligation"[Text Word] OR "SNL"[Text Word] OR "chronic compression dorsal root ganglion"[Text Word] OR "CCD"[Text Word] OR "spared nerve injury"[Text Word] OR "SNI"[Text Word]

#3 #1 AND #2

**2. Search Strategy for Web of Science**

#1 TS=("MicroRNA*" OR "mir*" OR "micro RNAs" OR "micro RNA" OR "micro-RNAs" OR "micro-RNA")

#2 TS=("sciatica*" OR "ischial*" OR "sciatic neuropathy" OR "sciatic pain" OR "sciatic nerve palsy" OR "sciatic nerve diseases" OR "sciatic neuritis" OR "chronic constriction injury" OR "CCI" OR "partial sciatic nerve injury" OR "PNI" OR "spinal nerve ligation" OR "SNL" OR "chronic compression dorsal root ganglion" OR "CCD" OR "spared nerve injury" OR "SNI")

#3 #1 AND #2

Timespan=All years. Databases=SCI-EXPANDED, SSCI, A&HCI, CPCI-S, CPCI-SSH.

**3. Search Strategy for EMBASE:**

#1 'MicroRNA'/exp OR 'mir*':ab,ti OR 'microRNAs':ab,ti OR 'microRNA':ab,ti OR 'micro RNAs':ab,ti OR 'micro RNA':ab,ti OR 'micro-RNAs':ab,ti OR 'micro-RNA':ab,ti

#2 'sciatica'/exp OR 'sciatica*':ab,ti OR 'ischial*':ab,ti OR 'sciatic neuropathy':ab,ti OR 'sciatic pain':ab,ti OR 'sciatic nerve palsy':ab,ti OR 'sciatic nerve diseases':ab,ti OR 'sciatic neuritis':ab,ti OR 'chronic constriction injury':ab,ti OR 'CCI':ab,ti OR 'partial sciatic nerve injury':ab,ti OR 'PNI':ab,ti OR 'spinal nerve ligation':ab,ti OR 'SNL':ab,ti OR 'chronic compression dorsal root ganglion':ab,ti OR 'CCD':ab,ti OR 'spared nerve injury':ab,ti OR 'SNI':ab,ti

#3 #1 AND #2

**4. Search Strategy fo****r CINAHL (EBSCO)**

S1 TX("MicroRNA*" OR "mir*" OR "micro RNAs" OR "micro RNA" OR "micro-RNAs" OR "micro-RNA")

S2 TX("sciatica*" OR "ischial*" OR "sciatic neuropathy" OR "sciatic pain" OR "sciatic nerve palsy" OR "sciatic nerve diseases" OR "sciatic neuritis" OR "chronic constriction injury" OR "CCI" OR "partial sciatic nerve injury" OR "PNI" OR "spinal nerve ligation" OR "SNL" OR "chronic compression dorsal root ganglion" OR "CCD" OR "spared nerve injury" OR "SNI")

S3 S1 AND S2
