# Supplementary figures and images for: Network and pathway‐based analysis of microRNA role in neuropathic pain in rat models
Source: J Cell Mol Med. 2019 May 8;23(7):4534–44. doi: 10.1111/jcmm.14357 (PMC6584487; doi:10.1111/jcmm.14357)

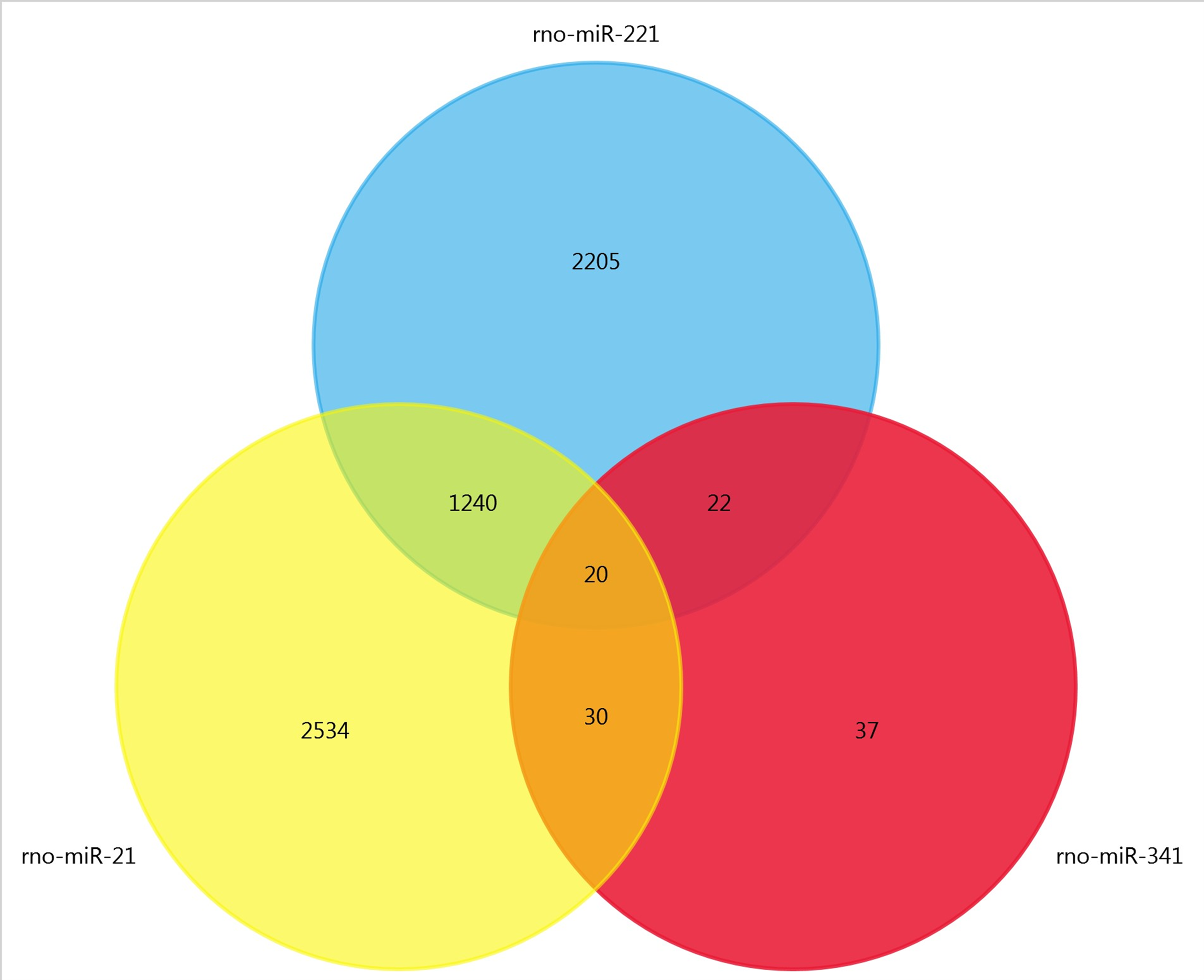

Supplement: Supplementary file 2 [file JCMM-23-4534-s002.tif]

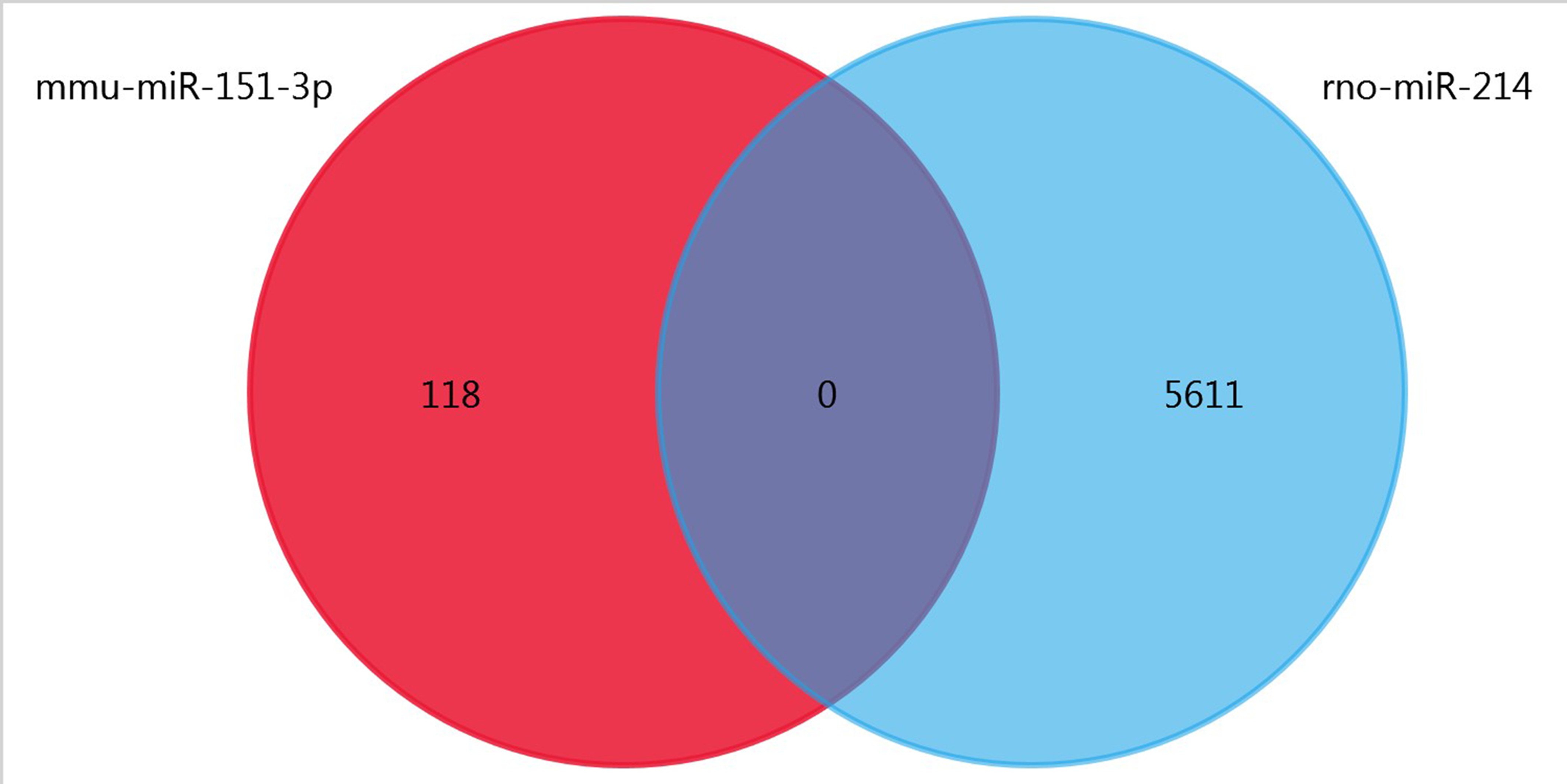

Supplement: Supplementary file 3 [file JCMM-23-4534-s003.tif]
