# Supplementary material for: Network and pathway‐based analysis of microRNA role in neuropathic pain in rat models
Source: J Cell Mol Med. 2019 May 8;23(7):4534–44. doi: 10.1111/jcmm.14357 (PMC6584487; doi:10.1111/jcmm.14357)
Supplement: Supplementary file 4 [file JCMM-23-4534-s004.docx]

**Supplementary Table S1 KEGG Analysis**

| Pathway description | Enriched genes | False discovery rate ( *p*-value) | Enrichment score (–log10 [*P*-value]) |
| --- | --- | --- | --- |
| Axon guidance | 6 | 0.0393 | 1.4056 |
| Circadian entrainment | 5 | 0.0393 | 1.4056 |
| Insulin secretion | 5 | 0.0393 | 1.4056 |

Abbreviation: Log10[P-value] is the logarithm of the P-value, and P<0.05 was considered significant.
