# Supplementary material for: Network and pathway‐based analysis of microRNA role in neuropathic pain in rat models
Source: J Cell Mol Med. 2019 May 8;23(7):4534–44. doi: 10.1111/jcmm.14357 (PMC6584487; doi:10.1111/jcmm.14357)
Supplement: Supplementary file 5 [file JCMM-23-4534-s005.docx]

**Appendix S1** Search strategies for all databases.

**Figure S1** Venn diagram analysis for rno-miR-221, rno-miR-21, and rno-miR-341.

**Figure S2** Venn diagrams analysis for rno-miR-214 and mmu-miR-151-3p.

**Table S1** KEGG analysis. KEGG, Kyoto Encyclopedia of Genes and Genomes.
